# The microtubule stabilizer patupilone counteracts ionizing radiation-induced matrix metalloproteinase activity and tumor cell invasion

**DOI:** 10.1186/1748-717X-8-105

**Published:** 2013-04-30

**Authors:** Polina Furmanova-Hollenstein, Angela Broggini-Tenzer, Matthias Eggel, Anne-Laure Millard, Martin Pruschy

**Affiliations:** 1 Laboratory for Molecular Radiobiology, University Hospital Zurich, Rämistrasse 100, Zurich, 8091, Switzerland; 2 Division of Infectious Diseases and Hospital Epidemiology, University Hospital Zurich, Zurich, Switzerland; 3 Institute for Cell Biology, University Berne, Berne, Switzerland

**Keywords:** Matrix metalloproteinase, Tissue inhibitor of metalloproteinases, Ionizing radiation, Microtubule stabilizing agent, Patupilone

## Abstract

**Background:**

Ionizing radiation (IR) in combination with microtubule stabilizing agents (MSA) is a promising combined treatment modality. Supra-additive treatment responses might result from direct tumor cell killing and cooperative indirect, tumor cell-mediated effects on the tumor microenvironment. Here we investigated deregulation of matrix metalloproteinase (MMP) activity, as an important component of the tumor microenvironment, by the combined treatment modality of IR with the clinically relevant MSA patupilone.

**Methods:**

Expression, secretion and activity of MMPs and related tissue inhibitors of metalloproteinases (TIMPs) were determined in cell extracts and conditioned media derived from human fibrosarcoma HT1080 and human glioblastoma U251 tumor cells in response to treatment with IR and the MSA patupilone. Treatment-dependent changes of the invasive capacities of these tumor cell lines were analysed using a Transwell invasion assay. Control experiments were performed using TIMP-directed siRNA and TIMP-directed inhibitory antibodies.

**Results:**

Enzymatic activity of secreted MMPs was determined after treatment with patupilone and irradiation in the human fibrosarcoma HT1080 and the human glioblastoma U251 tumor cell line. IR enhanced the activity of secreted MMPs up to 2-fold and cellular pretreatment with low dose patupilone (0.05-0.2 nM) counteracted specifically the IR-induced MMP activity. The cell invasive capacity of HT1080 and U251 cells was increased after irradiation with 2 Gy by 30% and 50%, respectively, and patupilone treatment completely abrogated IR-induced cell invasion. Patupilone did not alter the level of MMP expression, but interestingly, the protein level of secreted TIMP-1 and TIMP-2 was lower after combined treatment than after irradiation treatment alone. Furthermore, siRNA depletion of TIMP-1 or TIMP-2 prevented IR-mediated induction of MMP activity and cell invasion.

**Conclusions:**

These results indicate that patupilone counteracts an IR-induced MMP activation process by the reduction of secreted TIMP-1 and TIMP-2 proteins, which are required for activation of MMPs. Since IR-induced MMP activity could contribute to tumor progression, treatment combination of IR with patupilone might be of great clinical benefit for tumor therapy.

## Background

Interference with microtubular (MT) function represents a clinically relevant anticancer strategy, as demonstrated by the use of taxanes for the treatment of a wide variety of human malignancies [1-3]. Epothilones are nontaxoid MSAs of bacterial origin, which share the same binding site on β-tubulin with taxanes [4,5] and also promote tubulin polymerization and stabilization of already formed MTs [6]. Members of the epothilone family, including patupilone (epothilone B), have been tested in multiple clinical trials [7-13], Combined application of patupilone with ionizing radiation (IR) is a promising combined treatment modality for cancer therapy, as previously investigated in our laboratory in different tumor models such as lung and colon adenocarcinoma and medulloblastoma. The combined treatment modality results in strong supra-additive growth inhibition of murine tumor xenografts [14-16]. Interestingly though, combined cytotoxicity is much less pronounced on the cellular level *in vitro,* indicating that an additional effect occurs on the level of the tumor microenvironment. Further investigations revealed that patupilone treatment inhibits VEGF-secretion from the tumor cells thereby contributing to the supra-additive cytotoxicity of the combined treatment modality observed *in vivo*[14].

Microtubule interference with MSA could deregulate the secretion of biologically active factors [14,17,18]. We recently demonstrated that patupilone counteracts stress-induced VEGF expression and secretion from patupilone-sensitive tumor cells [19]. Furthermore, MSA could disturb the secretion of matrix metalloproteinase thereby affecting the tumor microenvironment (see below) [20-22].

Matrix metalloproteinases (MMPs) are zinc-dependent endopeptidases with a broad variety of substrates including components of the extracellular matrix and growth factors, such as the transforming growth factor β (TGF- β). MMPs play an important role in cell survival, apoptosis, angiogenesis, matrix remodelling and metastasis and are regulated at several levels including transcriptional and posttranslational mechanisms and by endogenous tissue inhibitors of metalloproteinases (TIMPs) [23-25]. TIMPs, a family of proteins containing 4 members, TIMP1-4, inhibit MMP-activity by binding to the catalytic domain of active MMPs. However, TIMP-2 is also known to be a co-activator of MMP-2 zymogen in an MT1-MMP (MMP-14)-dependent proMMP-2 activation process [26]. Additionally, TIMPs have MMP independent biological functions, including mitogenic [27] and pro- and anti-apoptotic functions [28,29]. MMPs and TIMPs are secreted by tumor-associated stromal and cancer cells and are important components of the tumor microenvironment. Secretion of MMPs and TIMPs is partially executed in vesicles along the MT-cytoskeleton and is therefore a potential target for MSAs. Overexpression of MMPs and TIMPs is associated with a poor survival prognosis for almost all tumor types [23]. In preclinical and clinical studies, IR was demonstrated to upregulate MMPs [30,31], and enhanced levels of MMPs were detected in patients with lung and breast cancer after radiotherapy [32]. Thus, IR might contribute to a potential prometastatic effect.

Here we investigated the effect of patupilone and IR on MMP function *in vitro*. We demonstrated that patupilone inhibits both IR-induced MMP activity and IR-induced cell invasion of human fibrosarcoma and glioma cell lines. Furthermore, we analysed the mechanisms of the inhibitory effect of patupilone and demonstrated that patupilone counteracts IR-upregulated MMP-activity by interfering with TIMP-1 and TIMP-2 secretion.

## Methods

### Cell culture

The human fibrosarcoma cells HT1080 (ATCC No CCL-121) were grown in IMDM media. Human glioma cells U251 was cultured in DMEM media. Media was supplemented with 10% (v/v) FCS and 1% (v/v) penicillin-streptomycin. All cell culture media and supplements were obtained from Gibco (Invitrogen). Cells were grown at 37°C in a 5% CO_2_ humidified atmosphere. For control of identity, all cells throughout the course of the studies were monitored for cell morphology and growth pattern. Cells were cultured for not more than 10 passages.

To prepare conditioned cell culture media (CM) cells were pretreated with or without patupilone for 24 h and sham-treated or irradiated with the indicated doses of IR. The cell culture media was discarded 1 h after irradiation and cells were incubated for an additional 24 h in serum-free Ultraculture medium (Lonza, Fischer Scientific). Conditioned cell culture medium was collected, centrifuged at 10’000 g for 5 min at 4°C and supernatants were stored at −80°C.

### Transfection

Transfection was performed using Lipofectamine 2000 (Invitrogen). siRNAs for downregulation of human TIMP-1 (GenBank BC000866.1) and human TIMP-2 (GenBank BC052605.1) were synthesized by Microsynth (Switzerland). siRNA against firefly luciferase (siLuc) was used for control transfection. siRNA sequences (5′-3′):

siTIMP-1#1 GAUGUAUAAAGGGUUCCAAdTdT [33]

siTIMP-2#1 GGCACAUUAUGUAAACAUAdTdT [33]

siLuc CGTACGCGGAATACTTCGAdTdT (Microsynth).

siRNAs were designed using Microsynth siRNA Design Tool.

### Treatment modality

Irradiation was performed at room temperature using an Xstrahl 200 kV X-ray unit (Gulmay Medical) at 1 Gy/min. Patupilone was provided by Novartis Pharma AG (Basel, Switzerland).

### Clonogenic survival assay

Clonogenic survival assay was performed as described previously [14]. 24 h after plating, cells were treated with increasing concentrations of patupilone and irradiated 24 h later. Colonies were allowed to grow under normal cell culture conditions for 7–10 days before fixation with methanol/acetic acid (75%/25% v/v) and staining with 2% crystal violet. Colonies with more than 50 cells were counted manually. For the determination of the synergistic effect, the earlier described model of synergy was used [34].

### MMP activity assay

MMP activity in the CM was determined using a FRET-based MMP activity assay (SensoLyte MMP-2 assay kit, AnaSpec). According to the kit-description the peptide substrate can be cleaved by multiple MMPs and was used for the determination of pooled MMP activity. The substrate cleavage reaction was performed according to the manufacture’s instructions. The fluorescent signal was measured in a Tecan GENios spectrophotometer every hour for 14 hours. The acquired signal was adjusted to the cell number and presented as the initial velocity of the substrate cleavage reaction (V_0_), relative to control. V_0_ was determined as a slope of the curve – the fluorescent signal plotted versus time – using linear regression (GraphPad Software, Inc).

### Cell invasion

Transwell inserts (6.5 mm, 8 μm pores, Costar) were coated with Matrigel™ (Becton Dickinson). Complete cell culture media was used as attractant for migrating cells. 5’000 cells/insert for HT1080 cells and 8’000 cells/insert for U251 cell were seeded in 200 μL of serum-free media. Patupilone was added to cells at the moment of seeding and cells were irradiated 4 h thereafter. Cells were allowed to migrate for 24 h. For quantification, cells from the upper side of the insert were scraped away, inserts were fixed in Methanol/Acetic acid (75%/25%, v/v) and stained with DAPI. Invaded cells were counted manually under a fluorescent microscope or using the software (Imaris, Bitplane). Each experiment was carried out in quadruplicates.

### Quantitative real time PCR (qRT-PCR)

The total mRNA was isolated with RNeasy Plus Mini Kit (Qiagen). Reverse transcriptase reaction was conducted with 1 μg of mRNA with High Capacity cDNA Reverse Transcription Kit (Applied Biosystems). qRT-PCR was performed with Fast SYBR Green Master Mix on 7900H PCR Instrument (Applied Biosystems). Data were analysed using RQ-manager software (Applied Biosystems) by employing ΔΔCt method. The following primers were used:

MMP-1 F: TGTGGACCATGCCATTGAGAA, R: TCTGCTTGACCCTCAGAGACC;

MMP-2 F: CTTCCGTCTGTCCCAGGAT, R: CCCCAT AGAGCTCCTGAATG;

MMP-3 F: GGGCCATCAGAGGAAATG, R: CACGGTTGGAGGGAAACCTA;

MMP-9 F: GGCCACTACTGTGCCTTTGAG, R: GATGGCGTCGAAGATGTTCAC;

MMP-14 F: TGGAGGAGACACCCACTTTGA, R: GCCACCAGGAAGATGTCATTTC;

TIMP-1 F: AGTGGCACTCATTGCTTGTG, R: TTTTCAGAGCCTTGGAGGAG;

TIMP-2 F: TTTTGCAATGCAGATGTAGTGAT, R: TCCTTCTCACTGACCGCTTT;

18srRNA F: ATGGCCGTTCTTAGTTGGTG, R: CGCTGAGCCAGTCAGTGTAG;

TBP F: CGGCTGTTTAACTTCGCTTC, R: TTCTTG GCAAACCAGAAACC.

### Western blot

Western blot was performed as described previously [35]. The following antibodies were used: anti-TIMP-1 and −2 (neutralizing antibodies, R&D systems), anti-β-actin (Sigma). Western blot films were scanned with transillumination mode and the bands was quantified by Quantity One 4.6 software (Bio-Rad Laboratories).

### Statistical analysis

Statistical analysis was performed using GraphPad Prism 3–5 software (GraphPad Software, Inc.). A comparison between the groups was performed using *t*-test. Results are plotted as mean ± SE; the level of significance was set at P < 0.05.

## Results

### Patupilone inhibits IR-induced matrix metalloproteinase activity

**Figure 1:**
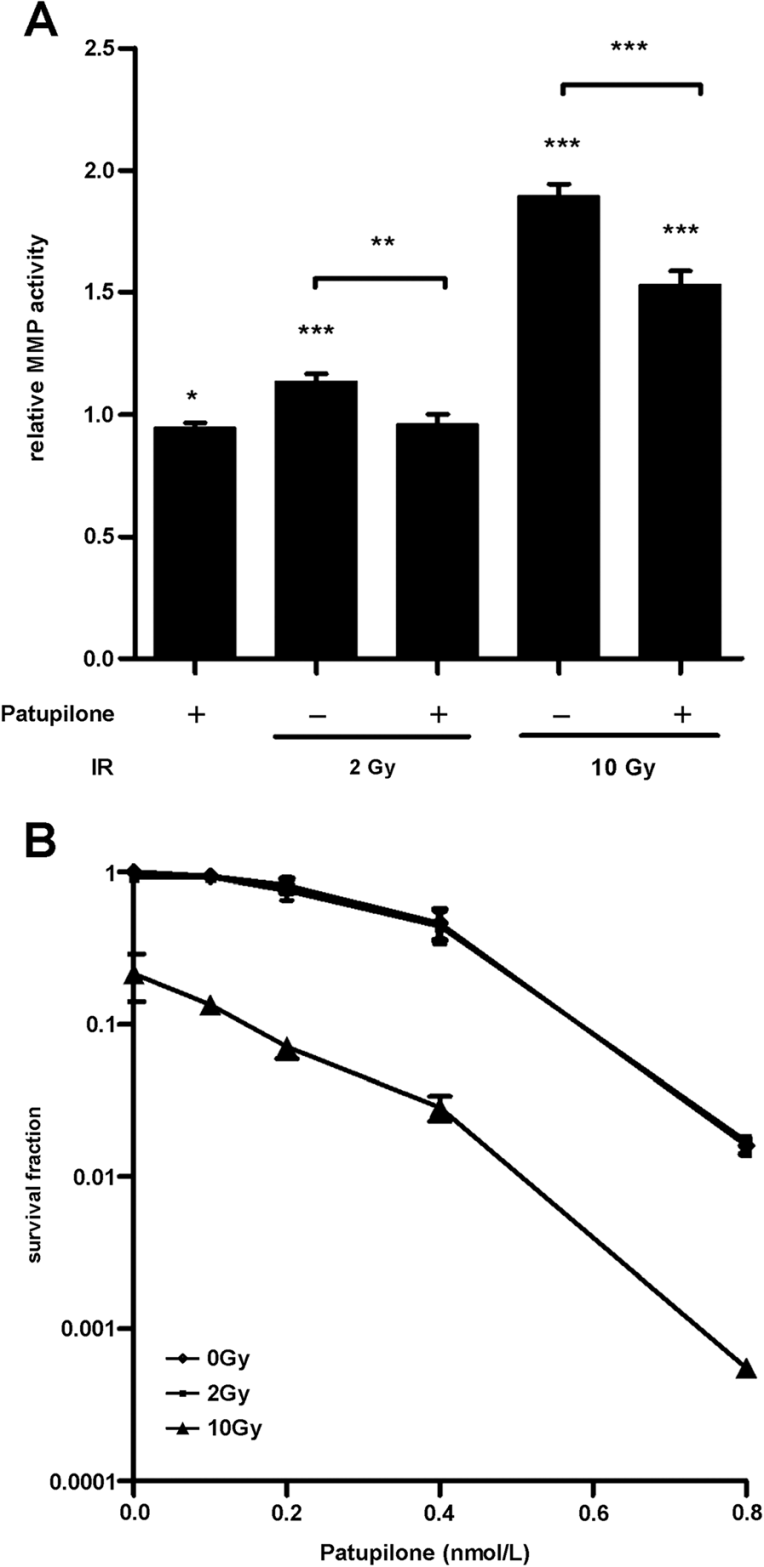
**Non-cytotoxic concentration of patupilone counteracts IR-enhanced MMP activity. A*,*** MMP activity was determined in the CM derived from HT1080 cells treated with 0.2 nM patupilone and indicated doses of IR. Cells were pretreated with or without patupilone for 24 h and sham-treated or irradiated with the indicated doses of IR. The cell culture media was discarded 1 h after irradiation and cells were incubated for additional 24 h in serum-free medium to obtain CM, n = 13. **B***,* clonogenic cell survival of HT1080 cells was determined after treatment with increasing doses of patupilone and IR, n = 3. *P < 0.05, **P < 0.01, ***P < 0.001.

To investigate the impact of IR and patupilone on secreted MMP activity, we determined the MMP activity in conditioned cell culture media (CM) derived from the human fibrosarcoma tumor cell line HT1080 24 h after treatment with IR and patupilone. A significant, dose-dependent increase of secreted MMP activity was observed after irradiation with 2 and 10 Gy of IR (1.15-fold; p < 0.01 and 2 fold; p < 0.001, respectively). Treatment with 0.2 nM patupilone alone only minimally decreased the MMP activity level (0.95 fold, p > 0.05) in comparison to the basal MMP activity level in CM derived from untreated control cells. Interestingly, pretreatment of cells with 0.2 nM patupilone completely abolished the IR-induced increase of MMP activity after 2 Gy and counteracted the irradiation-induced increase of MMP activity by 40% after 10 Gy of IR (p < 0.0001) (Figure 1A). A direct inhibitory effect of patupilone on MMPs could be excluded by addition of the MSA to CM, which did not affect MMP-activity (data not shown).

Long-term clonogenic survival of the HT1080 cells was determined after treatment with increasing doses of IR and patupilone (Figure 1B). Importantly low dose treatment with IR (2 Gy) or patupilone (0.2 nM), alone did not reduce clonogenicity of these fibrosarcoma cells. 10 Gy of IR reduced clonogenic cell survival of these radiation resistant cells to an SF of 0.3, and combined treatment with patupilone primarily induced an additive anti-clonogenic effect (Figure 1B). The proliferative activity of these HT1080 cells was only minimally reduced after treatment with patupilone (0.2 nM) alone and in combination with irradiation (10 Gy) (Additional file 1: Figure S1). Thus, patupilone significantly counteracted IR-induced MMP activity independent of a putative, antiproliferative effect of these treatment modalities.

### Patupilone does not regulate the expression of matrix metalloproteinases

**Figure 2:**
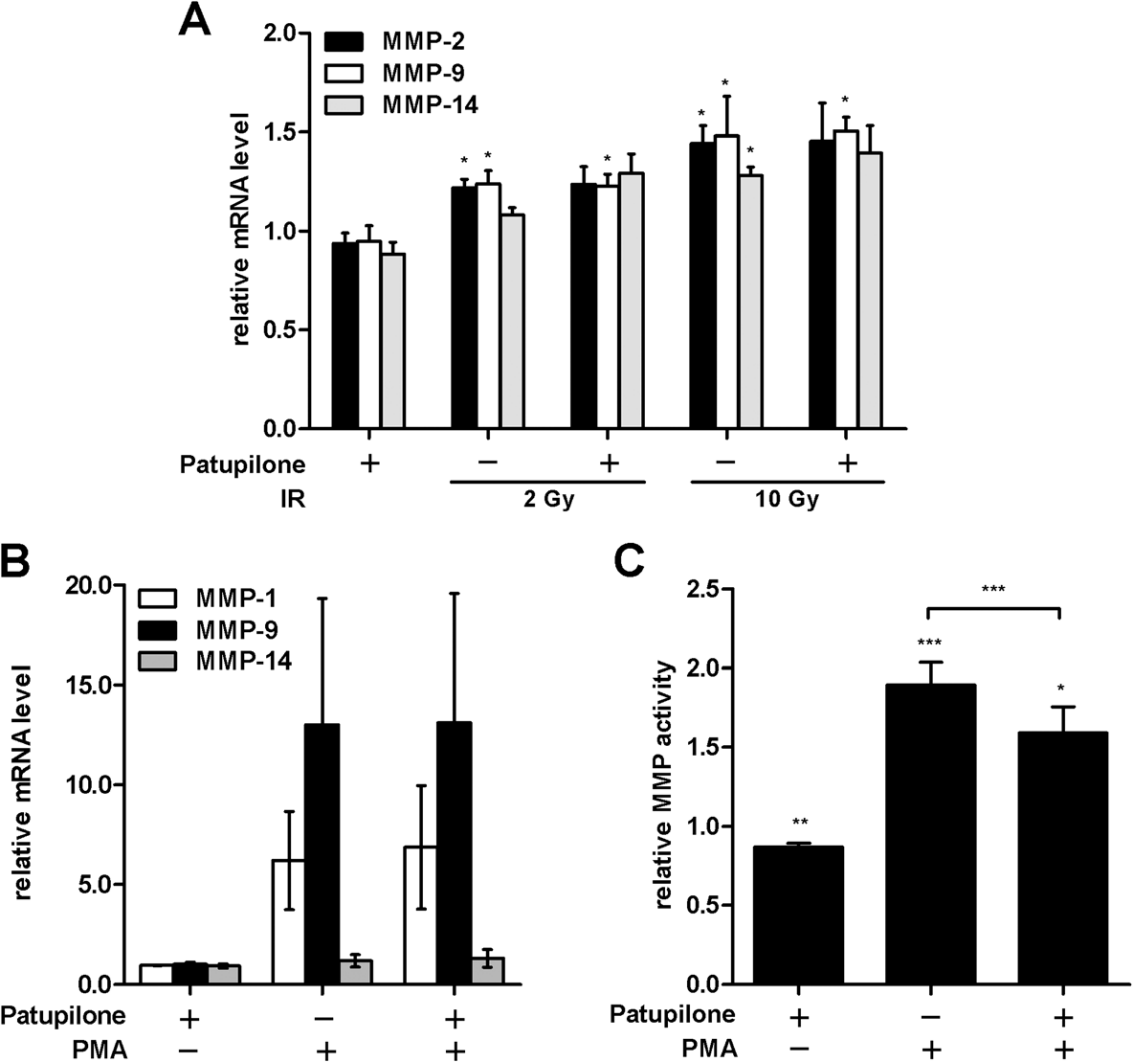
**Patupilone does not affect MMP transcription. A*,*** MMP mRNA levels in HT1080 cells were determined using qRT-PCR after treatment with 0.2 nM patupilone 24 h prior to IR. **B** and **C**, HT1080 cells were treated with 0.2 nM patupilone 24 h prior to treatment with PMA (0.65 mM). mRNA for qRT-PCR (**B**) and CM for determination of MMP activity (**C**) were isolated 18 h thereafter, n > 4. The fold increase in mRNA levels and MMP activity was normalized to the mRNA levels and MMP activity from untreated and sham-irradiated cells.*P < 0.05, **P < 0.01, ***P < 0.001.

To evaluate interference of patupilone and IR with MMP transcription, quantitative RT-PCR was performed with mRNA derived from HT1080 cells treated with 0.2 nM patupilone and IR (2 and 10 Gy), alone and in combination. A small but significant dose dependent-increase of MMP-2, -9 and −14- transcription (P = 0.002; P = 0.04-0.008; P = 0.0006, respectively) was induced by IR as determined 24 h after irradiation. Cellular pretreatment with patupilone altered neither the basal level of MMP transcription nor the level of IR-enhanced transcription (Figure 2A). We also assessed the mRNA levels of MMP-1 and MMP-3 but did not observe any significant changes under any treatment conditions (data not shown).

To determine interference of patupilone with MMP transcription by other known inducers of MMP-activity, cells were treated with phorbol-12-myristate-13-acetate (PMA), a strong transcriptional inducer of MMPs [36,37]. PMA-treatment upregulated MMP-9- and MMP-1-transcription by 13- and 5-fold, respectively. The proteolytic activity of MMPs, determined in the CM derived from PMA-treated cells, doubled after incubation with PMA (P = 0.0001). Pretreatment with patupilone did not counteract PMA-induced transcription (Figure 2B) but again diminished PMA-enhanced MMP-activity, as determined in CM derived from cells treated with PMA in combination with patupilone (P = 0.0001, Figure 2C).

Irradiation and patupilone-treatment might affect protein expression or secretion of matrix metalloproteinases. We therefore probed intra- and extracellular protein levels of MMPs (MMP-1, 2, 3, 9, 14) by western blotting and with gelatine zymography assays in case when no satisfactory antibody-based detection could be achieved. Since irradiation of HT1080 cells with 2 Gy only minimally increased MMP activity (Figure 1A) and mRNA levels (Figure 2A), experiments were performed with 10 Gy of irradiation. In cellular lysates derived from irradiated HT1080 cells only a minimal increase of MMP-9 protein level could be detected. On the other hand, in conditioned media, the protein level of all MMPs tested (MMP-2, 3) were slightly increased in response to irradiation (Additional file 2: Figure S2). These results correspond to the slight IR-induced increase of MMP-transcription (see above). However cellular pretreatement with patupilone altered neither the basal nor the IR-induced protein level of MMP-2, -3 and −9 in conditioned media (Additional file 2: Figure S2). Thus, the inhibitory, counteracting effect of patupilone on IR-induced MMP activity is not due to downregulation of MMP protein expression or secretion.

### Treatment with patupilone prevents the extracellular accumulation of the secreted TIMP-1 and TIMP-2 proteins

**Figure 3:**
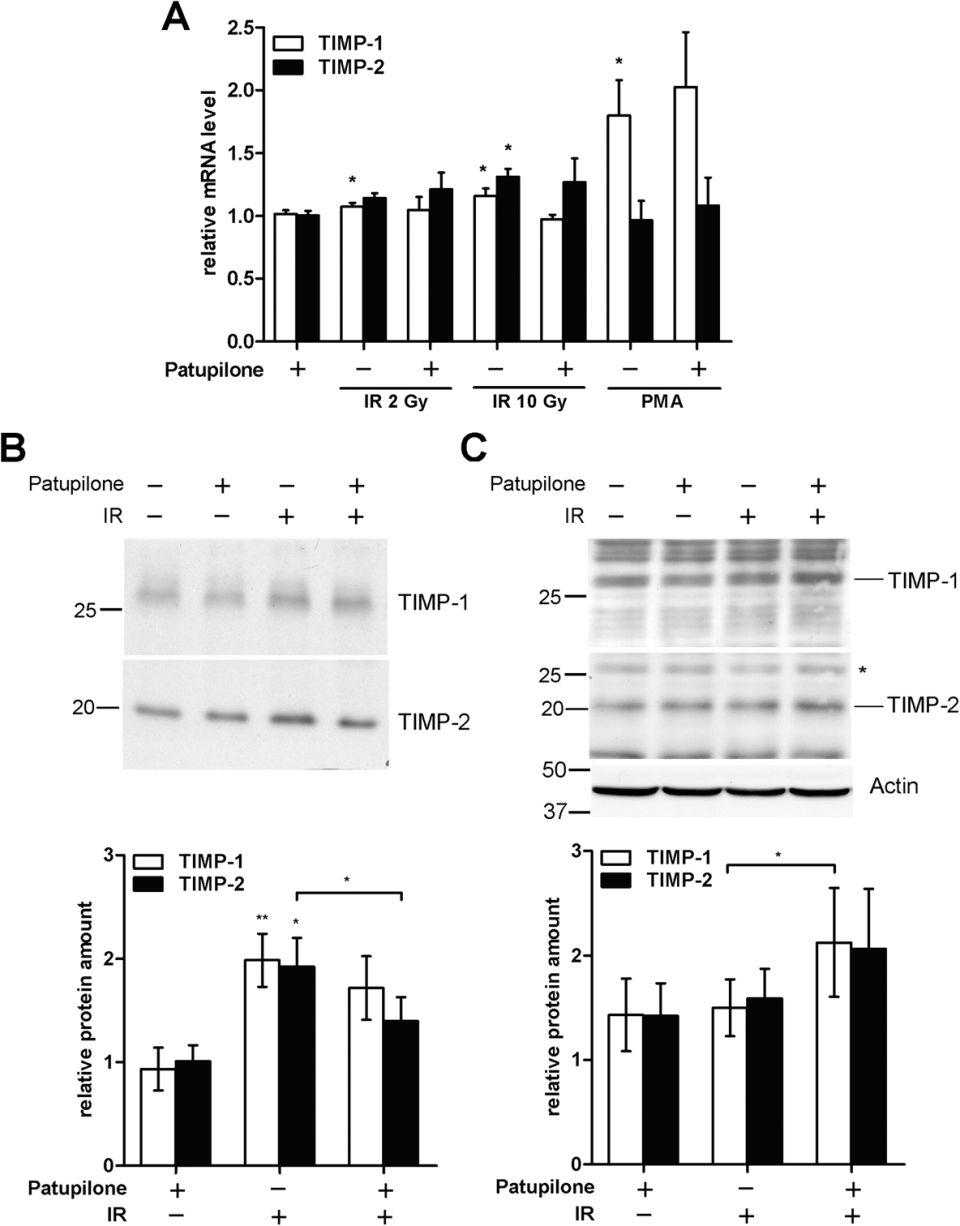
**TIMP-1 and TIMP-2 protein levels are regulated by IR and patupilone.** HT1080 cells were treated with patupilone (0.2 nM) and IR (2, 10 Gy) or PMA (0.65 mM). **A,** TIMP mRNA levels were determined using qRT-PCR, n > 4. **B** and **C**, protein levels of TIMPs were determined by western blotting in the CM (**B**) or cell lysate (**C**). Representative blots are shown; quantification was performed with at least 4 independent experiments. * indicates a TIMP-1 band, which was still detectable after washing and restaining of the membrane with TIMP-2 antibody. *P < 0.05, **P < 0.01.

MMP activity is regulated on multiple levels, including proteolytic activation, secretion and interaction with inhibitory factors [38]. In particular, MMP-activity is tightly controlled by the endogenous inhibitors TIMP-1 and TIMP-2 [25]. We therefore determined expression of the TIMPs after single and combined treatment with patupilone and IR. In response to increasing doses of IR only a minimal increase of TIMP-1 and TIMP-2 mRNA levels was observed. Patupilone pretreatment slightly reduced IR (10 Gy)-induced TIMP-1 mRNA-levels. Basal and IR-induced TIMP-2 mRNA levels were not affected by pretreatment with patupilone. Similar to MMP-expression, treatment of cells with PMA specifically induced transcription of TIMP-1 (up to 2-fold, P = 0.045) but not of TIMP-2, and again, patupilone did not interfere with PMA-upregulated transcription of this target gene (Figure 3A).

Next, we assessed protein levels of TIMP-1 and TIMP-2 in cell lysates and CM derived from the HT1080 fibrosarcoma cells, which were treated with IR and patupilone. In response to irradiation both TIMP-1 and TIMP-2 protein levels were increased in cell lysates and to a higher extent in CM derived from irradiated fibrosarcoma cells when compared to cell lysates and CM derived from control cells (Figure 3B, C). After treatment with patupilone alone, TIMP-1 and TIMP-2 protein levels were slightly elevated in cell lysates, while TIMP protein levels in CM did not change. Interestingly though, pretreatment with patupilone significantly counteracted the level of IR-enhanced TIMP-2-secretion (P = 0.02). A similar but not significant trend was also determined for TIMP-1 after treatment with patupilone in combination with IR (Figure 3B). Complementary to the counteracting effect of patupilone to IR-enhanced TIMP-protein levels in CM, TIMP protein levels in cell lysates were increased after combined treatment with IR and patupilone in comparison to cell lysates derived from cells treated with IR alone (P = 0.04 for TIMP-1; Figure 3C). These results indicate that patupilone does not affect basal and IR-induced TIMP-1 and TIMP-2 expression but rather interferes with IR-enhanced secretion of TIMPs into the CM. Eventually this will result in reduced TIMP-levels in CM in response to the combined treatment modality in comparison to treatment with IR alone.

### TIMP-1 and −2 are required to enhance matrix metalloproteinase activity by IR

**Figure 4:**
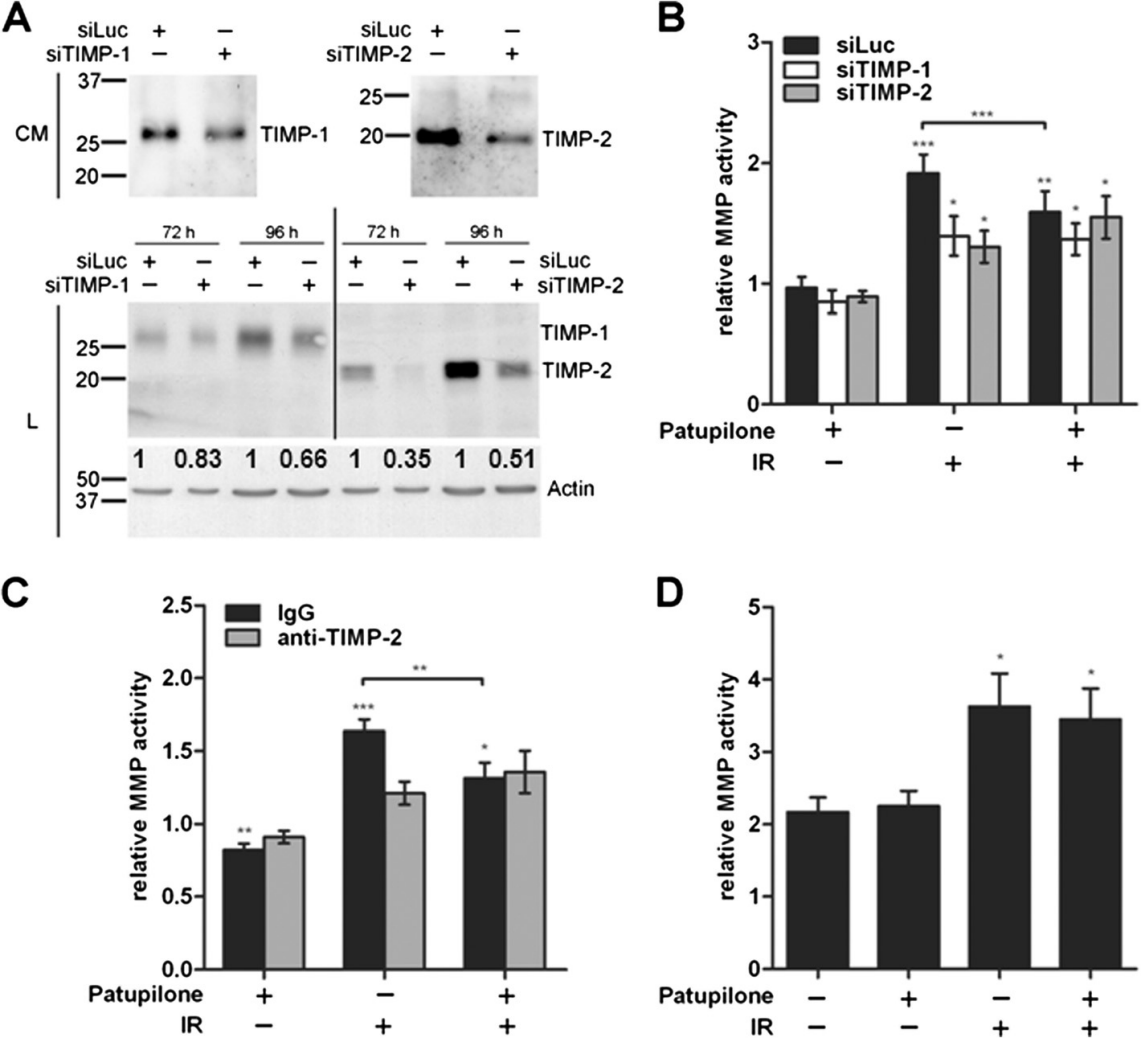
**TIMP protein levels determine IR-enhanced MMP activity. A*,*** effectiveness of protein downregulation by siRNA was assessed in the CM (96 h after transfection) and in cell lysate (L) by western blotting; representative blots are shown. A densitometric analysis of the TIMP-levels in the cell lysates, normalized to the loading control, was performed. **B**, MMP-activity in CM derived from siLuc-, siTIMP-1- and siTIMP-2-transfected HT1080 cells treated with 0.2 nM patupilone and 10 Gy IR, n ≥ 4. Cell treatment started 72 h after siRNA-transfection. **C**, HT1080 cells were incubated with anti-TIMP-2 or isotype control antibodies in parallel with patupilone (0.2 nM) and IR (10 Gy), n ≥ 4. **D**, CM, derived from HT1080 cells treated with patupilone (0.2 nM) and IR (10 Gy), was incubated with APMA for 1 h, n = 5. The fold increase in MMP activity was normalized to the activity in patupilone-untreated and sham-irradiated cells (**B, C**) and APMA-treated cells (**D**).*P < 0.05, **P < 0.01, ***P < 0.001.

To directly assess the role of TIMPs in the IR-dependent MMP activation process, TIMP-1 and TIMP-2 were downregulated in HT1080 cells with siRNA directed against the respective TIMP (Figure 4A). The cellular response to irradiation in control (siLuc)-transfected cells was similar to the cellular response in non-transfected cells (Figure 1A), leading to an IR-dependent increase of MMP activity in the CM thereof (1.9 ± 0.13 fold, P = 0.0005). Interestingly, TIMP-1 and TIMP-2-depletion significantly abolished IR-dependent upregulation of MMP-activity in the CM (P = 0.02), to the same extent as patupilone-pretreatment counteracted IR-dependent upregulation of MMP-activity. Pretreatment of TIMP-depleted cells with patupilone did not result in an additive, counteractive effect (Figure 4B).

Additional experiments were perfomed using control (goat IgG) and TIMP-2-directed neutralizing antibodies to confirm the putative regulatory effect of TIMP-2 on the IR-induced MMP-activity. These antibodies were supplemented to the cell culture media during the whole course of the treatment with IR and patupilone and to the serum free cell culture media after irradiation. MMP-activity in CM of control antibody-treated cells increased 1.6-fold in response to irradiation (P = 0.0002). TIMP-2-directed neutralizing antibodies significantly suppressed IR-dependent upregulation of MMP-activity (P = 0.007), to an extent as observed by siRNA-mediated TIMP-2 depletion or by patupilone-pretreatment prior to irradiation alone (see above). Pretreatment of TIMP-depleted cells with patupilone did not result in an additive, counteractive effect with TIMP-directed neutralizing antibodies (Figure 4C).

Thus, extracellular exposure to anti-TIMP-2-directed antibodies had the same neutralizing effect on IR-induced upregulation of MMP activity as siRNA-based depletion of TIMP-1 or TIMP-2, suggesting that TIMP-2 interferes with the MMP-activation process at the extracellular site. Unfortunately no experiments could be performed with neutralizing anti-TIMP-1 antibodies, due to the lack of satisfactory neutralizing activity (based on in vitro evaluation with recombinant TIMP-1 and MMPs; data not shown). Overall, these data indicate that patupilone counteracts IR-upregulated MMP-activity through interference with the TIMP-1 and TIMP-2 protein levels and that it is rather the activating than the inhibitory function of TIMP-1 and TIMP-2 that is involved in the IR-dependent enhancement of MMP-activity.

4-amino-phenylmercuric acetate (APMA) is an organomercuric activator of MMP zymogens, which will fully activate pro-MMPs present in the CM and independent of natural TIMP/MMP activation processes [39]. CMs derived from patupilone and IR-treated HT1080 cells were incubated with APMA (1mM) and MMP activity was determined thereafter. APMA treatment increased the level of MMP activity in all cell culture conditioned media samples (control, patupilone and/or irradiation treated) by 2.1+/−0.16-fold when compared to MMP activity in the corresponding APMA-untreated CMs (P < 0.004). No difference in MMP activity levels was observed between APMA-treated CMs derived from control and patupilone-treated cells. MMP activity in the APMA-incubated CM derived from irradiated cells was further enhanced, when compared to the MMP activity in the APMA-activated control CM (P = 0.02). Interestingly, the MMP activity levels in the APMA-incubated CM derived from irradiated cells and cells treated with the combined treatment modality did not significantly differ. Thus, the counteracting effect of patupilone on IR-induced MMP activity could not anymore be observed after incubation of the respective CMs with APMA (Figure 4D). These results corroborate that patupilone counteracts IR-induced MMP activation presumably on the level of pro-MMP activation processes, since the inhibitory effect of patupilone could no longer be observed when pro-MMPs in the CM were forced into an active state by APMA.

### Patupilone inhibits IR-induced cell invasion

**Figure 5:**
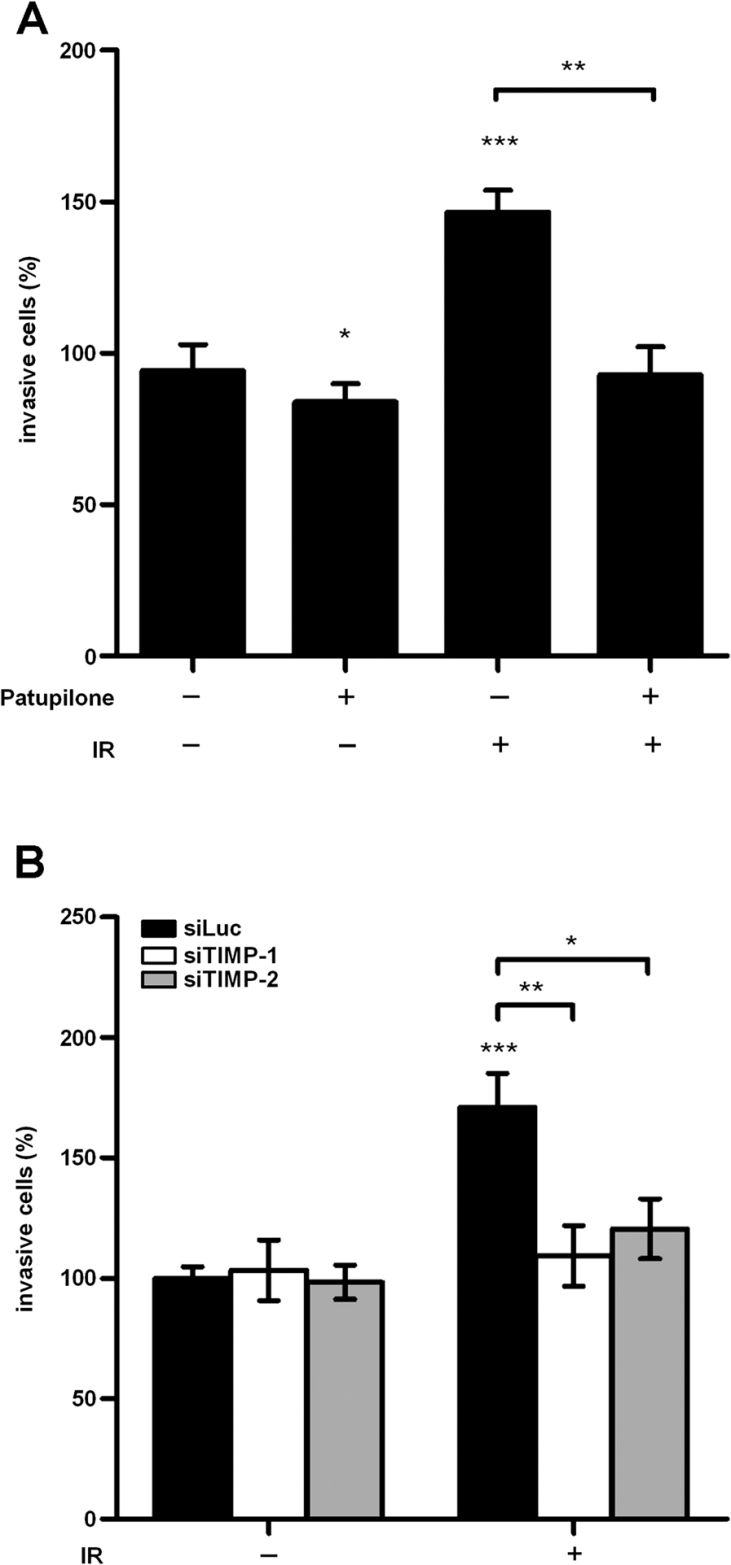
**IR-induced cell invasiveness is inhibited by patupilone and is dependent on TIMP protein level.** Invasiveness of the HT1080 cells was assessed in the Transwell invasion assay after treatment with patupilone (0.2 nM) and 2 Gy IR, n > 3 (**A**) as indicated in detail in the method section. **B**, HT1080 cells were transfected with siLuc, siTIMP-1 and siTIMP-2 prior to treatment with patupilone and IR, n = 5, followed by the invasion assay. *P < 0.05, **P < 0.01, ***P < 0.001.

Treatment-enhanced matrix metalloproteinase activity might directly translate into an increased invasive capacity of the target cells. Therefore, the invasive capacity of HT1080 cells was determined after treatment with patupilone and IR, alone and in combination, using a Transwell invasion assay. Treatment of cells with patupilone alone slightly decreased the basal invasion rate of HT1080 cells (to 83%, P = 0.03). Irradiation with 2 Gy resulted in an increased invasive capacity of HT1080 by 50%, (P < 0.0001) and most importantly, pretreatment of cells with patupilone completely abrogated IR-increased cell invasion (P = 0.0004; Figure 5A). The increase of cell invasion in response to IR could be attributed to enhanced MMP activity as the specific MMP inhibitor *N*-isobutyl-*N*-[4-methoxyphenylsulfonyl]glycyl hydroxamic acid (NNGH) inhibited cell invasion of irradiated cells (Additional file 3: Figure S3).

Depletion of TIMP-1 and TIMP-2 specifically counteracted IR-dependent upregulation of MMP-activity in the CM (Figure 4C, see above). Importantly depletion of TIMP-1 or TIMP-2 proteins with corresponding siRNAs also specifically abrogated IR-induced cell invasion (Figure 5B, P = 0.007 and 0.02 for siTIMP-1 and −2, respectively). Thus, these results suggest that IR-induced MMP activity translates into IR-enhanced cell invasion. Abrogation of IR-enhanced invasive capacity by patupilone is most probably due to interference with TIMP protein levels and subsequent pro-MMP activation processes.

**Figure 6:**
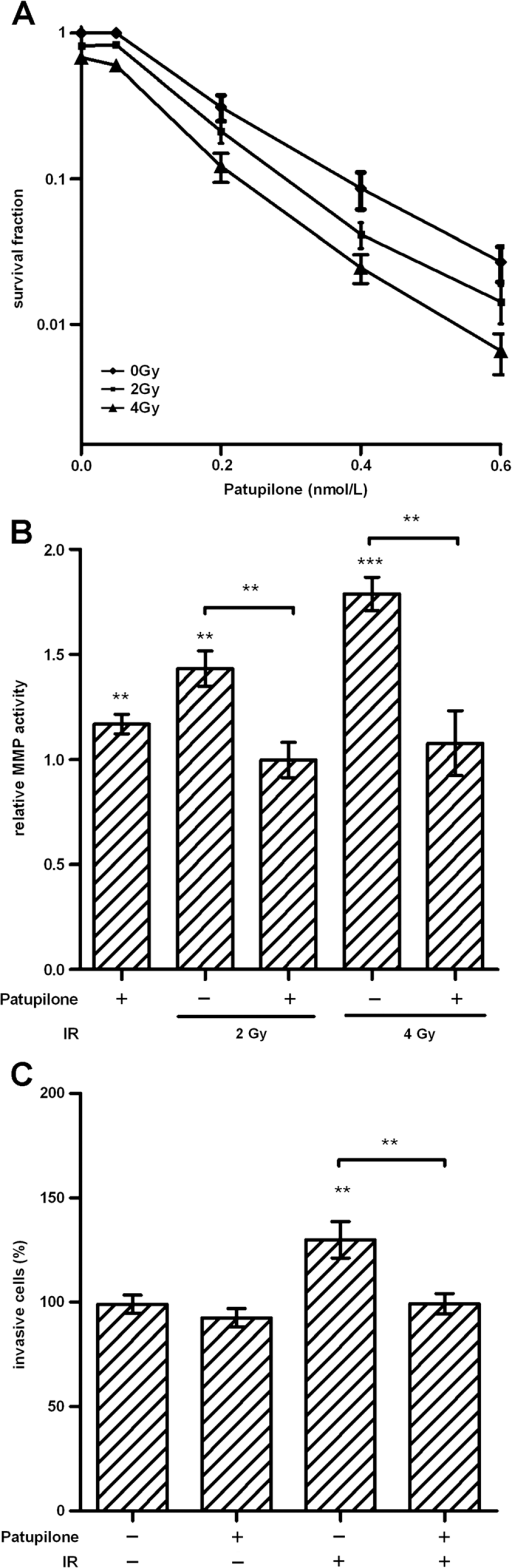
**Patupilone inhibits IR-induced MMP activity and cell invasion in human U251 glioma cells. A*,*** clonogenic cell survival of U251 cells was determined after treatment with patupilone and IR, similar to the experiments performed with the HT1080 cells, n = 3. **B**, MMP activity was measured in the CM derived from U251 cells treated with 0.05 nM patupilone and indicated doses of IR, n = 6. **C**, cell invasion was assessed using a Transwell invasion assay, after treatment with 0.05 nM of patupilone and 2 Gy of IR, n > 3. **P < 0.01, ***P < 0.001. MMP activity and cell invasion assays were performed similar to the experiments with the HT1080 cells.

To exclude a cell-line specific effect, matrix metalloproteinase activity and the invasive capacity were also determined in U251 human glioma cells after treatment with patupilone and IR alone and in combination. These experiments were performed at a concentration of patupilone (0.05 nM), that did not reduce clonogenicity of these cells and only minimally sensitized U251 cells to IR, and thus, corresponding to an equipotent concentration of patupilone as used for HT1080 cells (Figures 1B and 6A). Treatment with patupilone alone did not alter the basal level of MMP-activity in the CM derived from U251 cells. Irradiation with 2 and 4 Gy increased the MMP activity in the CM of U251 cells by 1.5- and 1.7-fold, respectively (P < 0.002), and pretreatment with patupilone completely abolished this increase (P < 0.004, Figure 6B). Treatment with 2 Gy of IR resulted in an increased invasive capacity of U251 cells by 30% (P = 0.0052), which was again reversed to basal level when cells were pretreated with patupilone (Figure 6C). Thus, the results obtained with the U251 human glioma cells correspond to the IR- and patupilone-dependent treatment responses as investigated in the HT1080 fibrosarcoma cell model.

## Discussion

Here we investigated the regulation of tumor cell-secreted matrix metalloproteinases in response to treatment with the promising combined treatment modality of IR with the clinically relevant MSA patupilone. To our best knowledge we demonstrated for the first time that a microtubule stabilizing agent counteracts IR-enhanced MMP activity and abrogates IR-induced tumor cell invasion. To uncover the molecular mechanism responsible for this counteracting effect, we determined MMP activity under different conditions. Patupilone did not alter IR-induced transcription of MMPs and TIMPs and did not influence intracellular and extracellular protein levels of MMPs. Interestingly though, patupilone counteracted the IR-enhanced cell invasive capacity via reduction of extracellular TIMP-1 and TIMP-2 protein levels, which are required for MMP-activation. The direct involvement of TIMPs in this patupilone-mediated counteracting effect was mechanistically demonstrated by the use of TIMP-neutralizing antibodies and in TIMP-depleted cells.

MMP inhibition represents an interesting strategy for combined treatment with IR, as successfully demonstrated by Kalinski et al. [40]. Previous investigations revealed that patupilone is a potent inhibitor of tumor cell proliferation under hypoxic and normoxic conditions, has anti-angiogenic properties, and radiosensitizes *in vivo* and *in vitro*[14,15,17,18,41-43]. In addition, as demonstrated in this study, patupilone inhibits IR-induced activity of secreted MMPs and IR-induced cell invasiveness. This aspect further increases the potency of patupilone as part of a combined treatment modality with IR and, probably, with other MMP-activating treatment modalities. At the same time these *in vitro* experiments corroborate our previous investigation on the level of VEGF-secretion that patupilone mechanistically interferes with IR-induced processes on the tumor cell level [14,19], which eventually strongly contribute to the supra-additive treatment response on the *in vivo* level.

Gene promoter regions of several MMPs contain putative binding sequences for stress response- and growth factor-induced transcriptional factors such as AP1 and Nf-κB ([38] and refs therein) and multiple reports confirmed that different stimuli activate MMPs and enhance TIMP protein levels via these pathways. IR was demonstrated to induce MMP-2 and MMP-9 and subsequently cell invasion via activation of the PI3K/Akt/Nf-κB pathways both *in vitro* and *in vivo*[30,31,40,44-47]. MSAs may disturb MMP activity and the regulatory function of TIMPs at the level of protein secretion, since intracellular transport of MMPs and TIMPs depends at least in part on an intact MT system [20,22]. For example, treatment with the MSA paclitaxel impaired secretion of MMP-2 and MMP-9 and significantly reduced the invasion of melanoma cells [21]. TIMPs can not only inhibit but also activate MMPs depending on the ratio of TIMPs and MMPs [48-50]. While TIMP-2 is relevant for processing of pro-MMP-2, the role of TIMP-1 in pro-MMP-9 activation has only been predicted based on the structural similarities of their complexes [51]. In our study, we corroborated that the TIMP/MMP ratio is of great importance for pro-MMP activation and may be deregulated in response to different treatment modalities, and further demonstrated that both TIMP-2 and TIMP-1 are important for MMP activation after radiation treatment. IR failed to enhance MMP activity to a large extent when the protein level of secreted TIMP-1 or TIMP-2 were downregulated by siRNA, neutralizing antibodies or patupilone treatment. Unfortunately and in part due to technical limitations, we could not identify which specific MMP is thereby affected and eventually responsible for the deregulated invasive capacity. Future studies will focus on a putative MMP redundancy and the specific molecular mechanisms of how MSAs and the microtubular dynamics interfere with the intracellular TIMP secretory processes.

MMPs play an important role in cancer progression, remodelling the extracellular matrix and processing multiple biologically active factors. MMPs are upregulated in multiple tumor types and their overexpression correlate with the aggressiveness of the disease. IR-enhanced cell invasion was mainly observed after irradiation with low doses of IR up to 2 Gy. This is most probably due to the concomitant IR-induced cytotoxicity at higher doses, which renders it difficult to differentiate between the two opposing processes. However, cell invasion induced by low radiation doses is of particular importance in the context of fractionated radiation schedules and sub-lethal irradiation of peripheral tumor cells of the radiotherapy treatment volume. Of particular interest is that the counteracting effect of patupilone could also be demonstrated in human glioma cells. Glioblastoma (or grade IV glioma) is one of the most treatment-resistant primary tumors in adults and overall survival still remains at a low rate of 14 months. A major reason for poor prognosis is extensive infiltration of surrounding brain tissue by tumor cells and the blood–brain barrier as an obstacle for the adequate delivery of cytotoxic agents [52]. Radiation therapy is a standard treatment for gliomas and to our best knowledge, we have demonstrated, for the first time, that patupilone can counteract IR-induced activity of secreted MMPs and a subsequent increase of the invasive capacity of glioma cells. The ability of patupilone to cross the blood–brain barrier and to retain in brain tissues [42] renders patupilone as a single treatment modality a very potent and promising chemotherapeutical agent for brain malignancies [11]. But in addition to the direct cytotoxic and radiosensitizing effect of patupilone, a combined treatment modality of IR with patupilone might minimize the risk for IR-induced glioma cell migration and dissemination, mediated by IR-upregulated MMPs [53,54]. However, beyond controlling tumor cell dissemination and due to the complex MMP functions in cancer progression and treatment resistance, the combined treatment modality of IR and patupilone might be of a great clinical benefit also for other malignancies.

## Conclusions

Previous investigations revealed additive cytotoxicity of IR with the microtubule stabilizing agent patupilone *in vitro* with a strong supra-additive effect *in vivo*. Here we mechanistically demonstrate *in vitro* that patupilone abrogates IR-induced metalloproteinase-activity and IR-induced invasive capacity of tumor cells. Since IR-induced MMP activity may limit tumor control and may even promote tumor progression, these results suggest an additional rationale to combine ionizing radiation with the microtubule interfering agent patupilone. Eventually these *in vitro* experiments corroborate our previous investigations that patupilone mechanistically interferes with IR-induced processes on the tumor cell level, which might strongly contribute to the supra-additive treatment response on the *in vivo* level.

## Competing interests

The authors declare that they have no competing interests.

## Authors’ contributions

PFH conducted all experiments and participated in the conception of the manuscript. ME participated in the conduction of parts of the experiments. A-LM participated in the conception of the research, the design of the experiments and interpretation of the data. ABT contributed to the revisions and the supplementary figures. MP conceived the research, directed all experiments and drafted the manuscript. All the authors have been involved in the drafting of the manuscript and have read and approved the final manuscript.

## Supplementary Material

Additional file 1: Figure S1 Proliferative activity of HT1080 cells 24 h after treatment with patupilone, IR and in combination. The proliferative activity of these HT1080 cells was only minimally and not significantly reduced after treatment with patupilone alone and in combination with irradiation. In case of the combined treatment the cells were treated with patupilone (0.2 nM) 24 h prior to IR (10 Gy). Proliferative activity was measured with the MTT-like Alamar Blue colorimetric proliferation assay 24 hours after IR. Mean +/− SD are shown of 3 independent experiments performed in triplicate. Click here for file

Additional file 2: Figure S2 mRNA and protein levels of MMPs after treatment. A, MMP-1 and −3 mRNA levels were determined using qRT-PCR in HT1080 cells treated with 0.2 nM patupilone 24 h prior to IR (10 Gy). RNA was isolated 18 h thereafter. B, The MMP protein levels in HT1080 cells were determined in the CM by western blotting (top) and by gelatine zymography (middle) and in the whole cell lysates by western blotting (bottom). The cells were treated with 0.2 nM patupilone 24 h before 10 Gy IR or application of 40 mg/ml PMA. 24 h thereafter, the cell lysates and CM were collected. N > 4. Click here for file

Additional file 3: Figure S3 The MMP inhibitor NNGH inhibits cell invasion. Cells were plated with NNGH (10 mM) 4 h prior to irradiation. The rate of invasion was evaluated 24 h after plating. The results are plotted as percentage of the invading cells relative to control. Mean +/− SE, n > 3, *P < 0.05, **P < 0.01, ***P < 0.001. Click here for file
