# Supplementary figures and images for: The microtubule stabilizer patupilone counteracts ionizing radiation-induced matrix metalloproteinase activity and tumor cell invasion
Source: Radiat Oncol. 2013 Apr 30;8:105. doi: 10.1186/1748-717X-8-105 (PMC3661365; doi:10.1186/1748-717X-8-105)

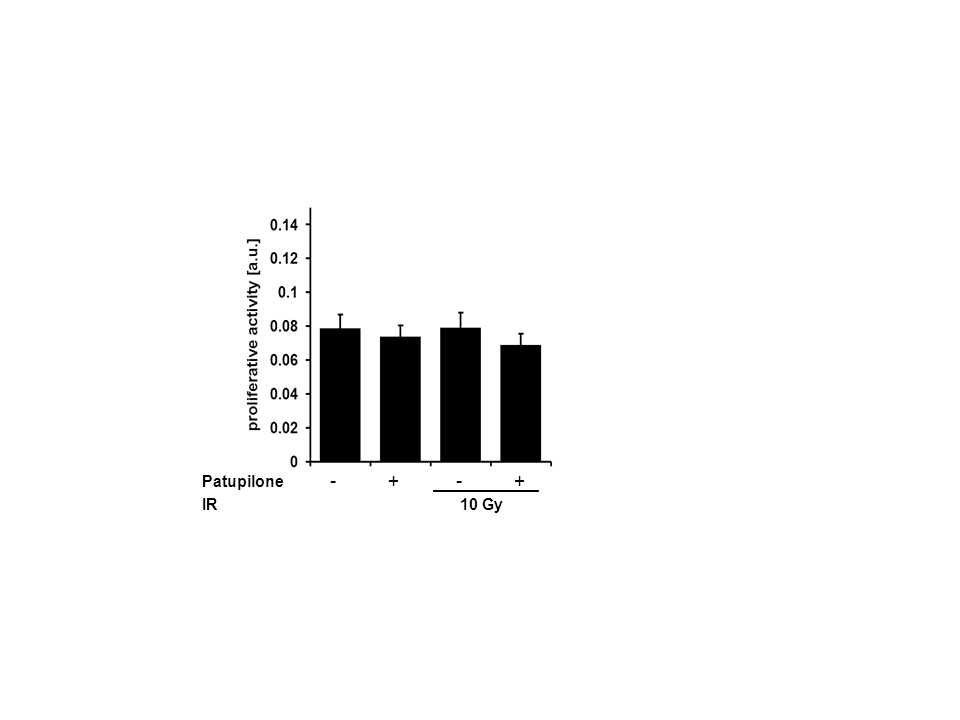

Supplement: Additional file 1: Figure S1 — Proliferative activity of HT1080 cells 24 h after treatment with patupilone, IR and in combination. The proliferative activity of these HT1080 cells was only minimally and not significantly reduced after treatment with patupilone alone and in combination with irradiation. In case of the combined treatment the cells were treated with patupilone (0.2 nM) 24 h prior to IR (10 Gy). Proliferative activity was measured with the MTT-like Alamar Blue colorimetric proliferation assay 24 hours after IR. Mean +/− SD are shown of 3 independent experiments performed in triplicate. [file 1748-717X-8-105-S1.tiff]

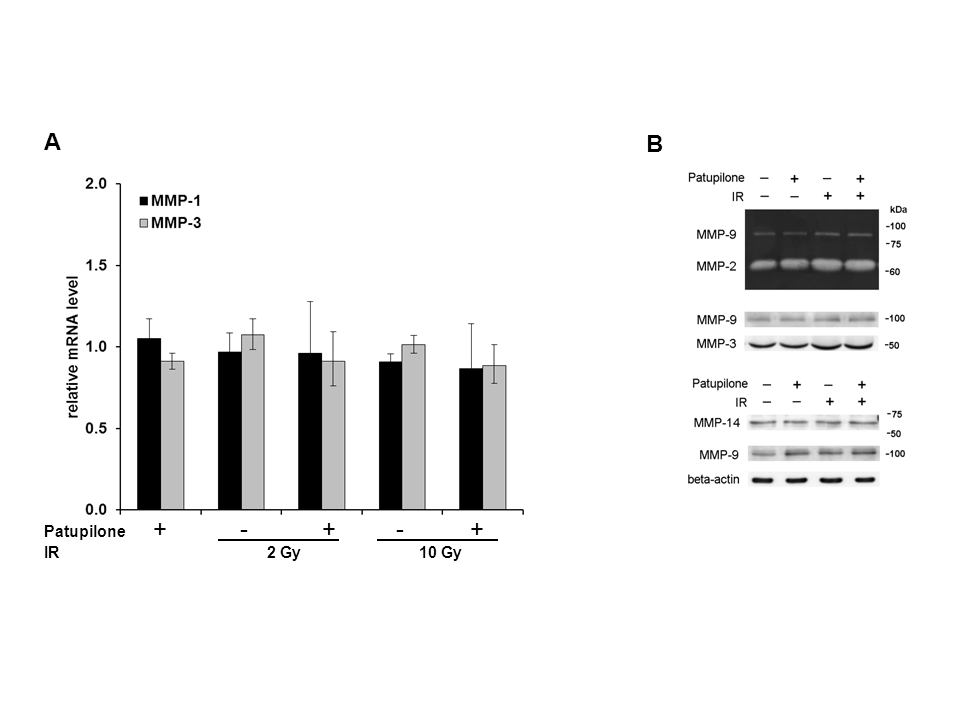

Supplement: Additional file 2: Figure S2 — mRNA and protein levels of MMPs after treatment. A, MMP-1 and −3 mRNA levels were determined using qRT-PCR in HT1080 cells treated with 0.2 nM patupilone 24 h prior to IR (10 Gy). RNA was isolated 18 h thereafter. B, The MMP protein levels in HT1080 cells were determined in the CM by western blotting (top) and by gelatine zymography (middle) and in the whole cell lysates by western blotting (bottom). The cells were treated with 0.2 nM patupilone 24 h before 10 Gy IR or application of 40 mg/ml PMA. 24 h thereafter, the cell lysates and CM were collected. N > 4. [file 1748-717X-8-105-S2.tiff]

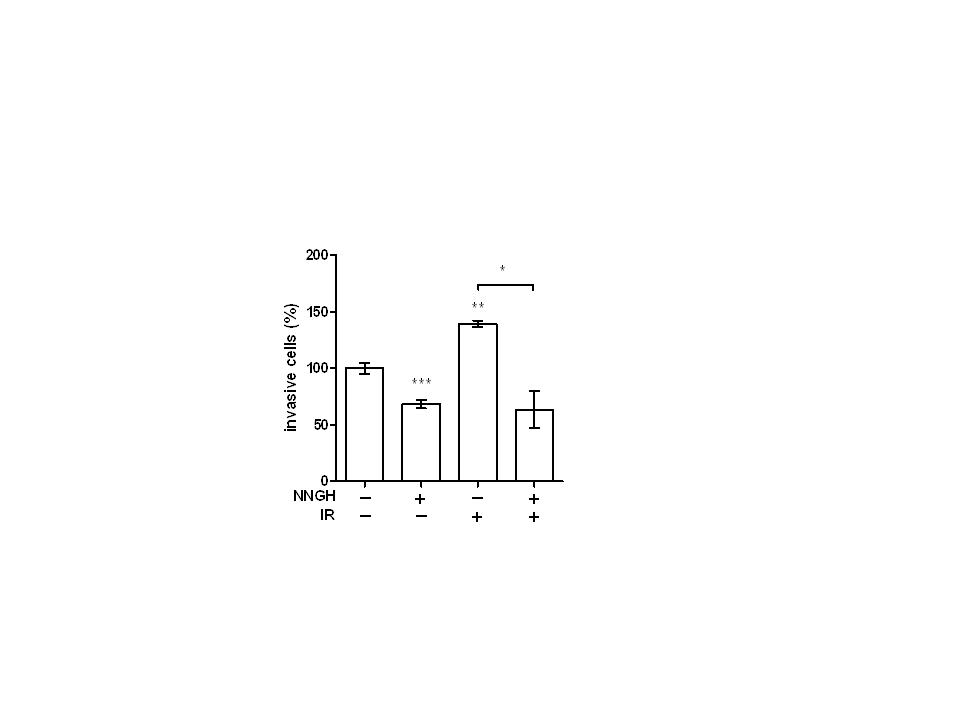

Supplement: Additional file 3: Figure S3 — The MMP inhibitor NNGH inhibits cell invasion. Cells were plated with NNGH (10 mM) 4 h prior to irradiation. The rate of invasion was evaluated 24 h after plating. The results are plotted as percentage of the invading cells relative to control. Mean +/− SE, n > 3, *P < 0.05, **P < 0.01, ***P < 0.001. [file 1748-717X-8-105-S3.tiff]
